# Constitutional Syphilis Communicated by the Father to the Fœtus Utero, the Mother Being Unaffected

**Published:** 1846-12

**Authors:** 


*Constitutional Syphilis communicated by the father to the fœtus in utero, the mother being unaffected.*—"Mr. Acton, Surgeon to the Islington Dispensatory, England, adduces three cases, in which constitutional syphilis in the father, was the cause of repeated abortions, and, subsequently, of infection of the fœtus born at the full period—the mother remaining throughout wholly free from disease.

A child nine weeks old, was brought to him, by its mother, on account of an eruption, chiefly papular, over the whole body. The voice was hoarse, and there was a slight discharge from the nose; the palms of the hands presented a scaly copper-colored eruption. Emaciation was less than is usually observed in children laboring under syphilis; but that peculiar earthy hue of the skin generally, was very evident. The mother stated, that she had been married four years—became pregnant soon after her marriage, and at the full time produced a dead child, the skin of which was dark colored, and peeled off on the slightest touch. During the following year she miscarried. On the occurrence of the third pregnancy, the child, that brought to Mr. A., was born at the full period and perfectly healthy. During the third week, spots were observed on the genital organs, and since then increased constantly in extent. No symptom of either primary or secondary disease, could be discovered in the mother. The father, shortly before his marriage, contracted chancres, was salivated, and secondary symptoms followed. He again took mercury, and believed himself perfectly cured at the time of his marriage. Denies having had any primary symptoms since—but has occasionally seen white spots on his mouth and tongue—has not remarked any spots on his body. There was nothing in his appearance to indicate syphilis, nor could any recent marks of infection be discovered. Mr. A. directed an ointment composed of ungt. hydrarg. nitrat. and spermaceti to be applied to the affected skin, and a powder containing two grains of hydrarg. c. creta, to be given at night. Within a month the child was free from disease, and had regained its healthy appearance.

Mr. A. gives an abridged account of two other cases of secondary syphilis in men, whose wives were free from all disease, but had miscarried. He remarks, that these cases furnish three instances of males affected with constitutional symptoms who marry and yet fail to communicate any disease to their wives, thus far corroborating our experiments that secondary symptoms are not capable of transmission from an affected male to a healthy female. They moreover make it probable that a male, thus affected, may so far exercise a morbid influence on the embryo, the result of cohabitation between him and a healthy female, as to cause a premature expulsion, or disease it so much, that, soon after birth, secondary symptoms will appear. The first case further induces this belief, that though syphilis may produce miscarriage, a healthy child can be subsequently born, although no mercury be given to either parent.

If it be true that the father can infect the fœtus without contaminating the mother, it justifies the surgeon in sparing her a course of mercury—a proceeding always injurious to the child, by deteriorating the milk—and may induce him to treat the child with some mild mercurial, without fear of its being reinfected by suckling the mother—thus offering additional evidence that the mother does not participate in the disease which the child inherits from the father." — *From Trans. College of Physicians, Philadelphia, April to Aug.*, 1846.

